# Seroprevalence and risk factors of brucellosis and the brucellosis model at the individual level of dairy cattle in the West Bandung District, Indonesia

**DOI:** 10.14202/vetworld.2021.1-10

**Published:** 2021-01-05

**Authors:** Yuli Yanti, Bambang Sumiarto, Tri Anggraeni Kusumastuti, Aprizal Panus, Sodirun Sodirun

**Affiliations:** 1 Graduate Student of Veterinary Science, Faculty of Veterinary Medicine, Gadjah Mada University, Yogyakarta 55281, Indonesia; 2 Epidemiology Laboratory, Disease Investigation Center of Subang, Subang, Indonesia; 3 Department of Veterinary Public Health, Faculty of Veterinary Medicine, Gadjah Mada University, Yogyakarta 55281, Indonesia; 4 Department of Social economic, Faculty of Livestock, Gadjah Mada University, Yogyakarta 55281, Indonesia; 5 Epidemiology Laboratory, Disease Investigation Center of Subang, Subang, Indonesia; 6 Disease Investigation Center of Subang, Subang, Indonesia

**Keywords:** brucellosis, modeling, prevalence, risk factors

## Abstract

**Background and Aim:**

Brucellosis is a zoonotic bacterial infectious disease. West Bandung is a center for dairy farming in West Java Province District and endemic for brucellosis. The aim of the study was to determine the prevalence, the associated risk factors, and the brucellosis model at the individual level of dairy cattle in the West Bandung District.

**Materials and Methods:**

The research was conducted through a cross-sectional study. The samples were collected from the serum blood of dairy cattle. Data obtained from the questionnaire were used to investigate risk factors. Multistage random sampling was applied as the sampling technique; therefore, a sample size of 540 cows was selected. The number of farms and cattle on each farm was calculated using a variant effect design of the farm as well as 108 farms was selected with five cattle samples per farm. The results in regard of the research sample acquisition in the West Bandung District included 588 dairy cows from 116 farms, exceeds the number of samples calculated (540 dairy cows and 108 farms). The rose Bengal test (RBT) and the complement fixation test (CFT) were performed for brucellosis testing. Data associated with brucellosis cases at the individual level of the dairy cattle were analyzed using descriptive statistics univariate, bivariate with Chi-square, and odds ratio (OR). Moreover, multivariate logistic regression was used for the analysis during modeling.

**Results:**

The results showed that the prevalence of brucellosis at the individual level in the West Bandung District was 5.10%. Risk factors associated with brucellosis in cattle included the history of abortion (p=0.000; OR=9.9), the history of placental retention (p=0.000; OR=6.6), the history of endometritis (p=0.000; OR=5.5), the history of stillbirth (p=0.043; OR=3.0), the history of pregnancy abortion age at 7-8 months (p=0.000; OR=15.2), and the history of pregnancy abortion at the age of 4-6 months (p=0.007; OR=3.8). The model of brucellosis in dairy cows was the following: = −3.2843+3.41033 the history of pregnancy abortion at the age of 7-8 months +2.54503 the history of pregnancy abortion at the age of 4-6 months +1.86185 age of cattle >2 years – 1.0469 Calving interval 12 months. The model showed the factors that were associated with brucellosis at the individual level of dairy cattle included the history of pregnancy abortion at the age of 7-8 months (β=+3.41033; OR=30.3), the history of pregnancy abortion at the age of 4-6 months (β=+2.54503; OR=12.7); age of cattle >2 years (β=+1.86185; OR=1.2), and Calving interval ≤12 months (β=−1.04691; OR=0.34).

**Conclusion:**

The results of this research showed that the prevalence of brucellosis at the individual level of dairy cattle in the West Bandung district was 5.10%. The risk factors could contribute to the increase of the brucellosis cases, that is, the history of pregnancy abortion at the age of 7-8, the history of pregnancy abortion at the age of 4-6 months, and the age of cattle >2 years. The risk factors can be decreased in the brucellosis cases, that is, calving intervals ≤12 months.

## Introduction

Brucellosis is considered to be one of the most important zoonoses in the world [1,2]. *Brucella abortus* is the main causative agent of brucellosis in cattle, triggering abortion, and infertility in adult animals [3,4]. *Brucella* species are facultative, intracellular Gram-negative bacteria with marked tropism for the pregnant reproductive tract of domestic animals. All *Brucella* species trigger persistent infection in the reticuloendothelial system in their natural hosts. The *Brucella* cell wall components consist of peptidoglycan, proteins, and outer membranes made of lipoproteins and lipopolysaccharides (LPS) in both fine strains (smooth), including *Brucella melitensis*, *B. abortus*, and *Brucella suiz*, as well as rough strains, including *Brucella canis*. LPS are responsible for the bactericidal effect in macrophage cells and are the determinant of *Brucella* bacterial virulence [5]. The clinical symptoms of brucellosis in cattle include abortion, infertility, stillbirth or weak birth, epididymitis, and orchitis in male animals [6,7] which can be followed by temporary infertility or permanent and decreased milk production [8]. Abortion usually occurs at the gestational age of 5-8 months (third trimester) [9,10]. Cattle infected with brucellosis can experience abortion 1-3 times that are subsequently followed by normal birth without symptoms of brucellosis, although they still excrete infectious vaginal fluid [11].

The occurrence of brucellosis in West Java Province, Indonesia, was previously reported to be quite high with a prevalence of 3.6% [12]. In contrast, the prevalence of brucellosis in West Bandung District can reach up to 7.5% [13]. The West Bandung District has a dairy farm with a fairly large cattle population in West Java Province. *Brucella* bacteria grow at a temperature between 20 and 37°C with an optimum of 34°C, the water temperature of 4°C, and in soil of 18°C. The climate in West Bandung District shows very similar characteristics. The transmission of brucellosis in cattle can occur horizontally from an infected cow to other vulnerable animals both on the individual level and in herds. Brucellosis can also be transmitted vertically from an infected female animal to the embryo or fetus during its stay in the uterus [10,14]. Female cattle are a source of transmission of brucellosis infection to other animals [15] which mainly occurs through the secretion of uterine fluid, placental tissue, fetus, infected cattle, or semen contaminated with *Brucella* [2,16].

Brucellosis risk factors can be categorized into animal-related, managerial, and environmental factors. Animal-related factors include age, species, the history of abortion, and placenta retention, as well as milking methods [17-19]. The management of farms is also an important risk factor in association with causing the disease. Environmental factors are mainly related to the agro-ecological location of animals in endemic or brucellosis-free locations [20,21]. Therefore, it is particularly important to know the prevalence and risk factors of brucellosis in association with dairy cattle which are useful for the control and management of the disease in the West Bandung District.

The study aimed to determine the brucellosis case model at the individual level of dairy cattle and find out the prevalence of brucellosis as well as investigate the risk factors associated with the disease in the West Bandung District, Indonesia.

## Materials and Methods

### Ethical approval and informed consent

Ethical approval for this study was obtained from the Animal Ethics Committee of the Faculty of Veterinary Medicine, Gadjah Mada University, Yogyakarta, Indonesia. The ethical clearance certificate number 0086/EC-FKH/Int/2019, dated July 26, 2019. Informed consent was obtained from all the participants prior to the study.

### Study period and location

**Figure-1:**
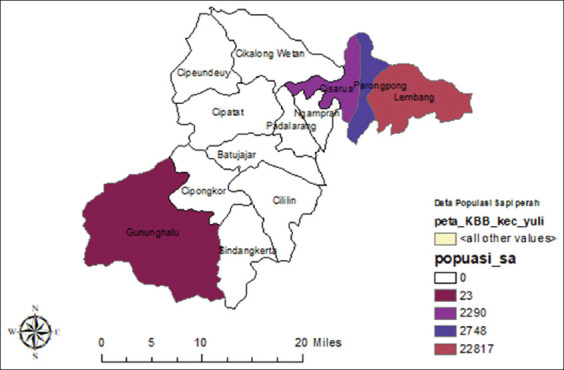
Map of dairy cattle population distribution in KBB [Source: Map prepared by the corresponding author].

The research was conducted from August 2019 to March 2020 in West Bandung District as one of the centers of dairy farming in West Java Province with 27,878 dairy cattle population, which is the most distributed in three subdistricts such as Lembang, Parongpong, and Cisarua (Figure-1).

### Study framework

A cross-sectional study was conducted to find out the prevalence of brucellosis and to investigate the risk factors associated with the cases of the disease in the West Bandung District. The calculation of the sample size was performed using the formula of Martin *et al*. [22] (n=4 PQ/L^2^) based on the prevalence of brucellosis in the West Bandung District in 2017 (7.5%) [13] with a maximum error of 5%, and a confidence level of 95%. Multistage random sampling was used as the sampling technique, so a sample size of 540 cattle was selected. The number of farms and cattle per farm was calculated using the design of farm variance effects with the formula (S^2^=P.Q/L^2^ with P=0.075; Q=1-P=0.925; L=0.050) based on which 108 farms were selected with five cattle from each farm. The results in regard of the research sample acquisition in the West Bandung District included 588 dairy cows from 116 farms, exceeds the number of samples calculated (540 dairy cows and 108 farms).

The research sample was taken from three districts with relatively large dairy populations, namely the Cisarua, Lembang, and Parongpong districts. Five milliliters blood sample was collected from each cattle through a jugular vein using a 10 mL syringe. The blood was left at room temperature for 24 h and the serum was harvested using cryovials. Subsequently, each cryovial containing the serum was labeled. Relevant risk factors, including age, breed, and sex, were also recorded simultaneously during blood collection. The collected serum sample was stored at −20°C until further testing by both rose Bengal plate test and complement fixation test (CFT).

### Questionnaires

Risk factors data were collected by asking farmers using a self-designed closed questionnaire which was tested for validity and reliability using the IBM SPSS Statistics for Windows version 20.0. The validity value of the questions was measured by the SPSS Correlate Person Instrument, while the reliability value was measured by the reliability analysis instrument. The question in the questionnaire was deemed to be valid as a result showed p<0.05 as well as high reliability with the Cronbach’s alpha value (0.872)>0.6 [23].

### Testing method

Serum samples were examined using rose Bengal test (RBT) and samples with RBT positive results were subsequently analyzed by the CFT test as the gold standard [24].

### Statistical analysis

In this study, the obtained data were analyzed by descriptive statistics, univariate, and bivariate using the Chi-square test and the odds ratio (OR), as well as multivariate analysis by the IBM SPSS Statistics for Windows version 20.0 [22,23] and the Eldridg Avenue Statistics analytical software version 8 [25]. In the meantime, the model was created using multivariate logistic regression analysis with a significance value of p=0.10, 95% confidence level. The created model was Y=α+β_1_X_1_+β_2_X_2_+.........+β_n_X_n_+e [25].

## Results

The results in regard to the research sample acquisition in the West Bandung District included 588 dairy cows from 116 farms.

### Prevalence of brucellosis in dairy cattle

The prevalence of brucellosis at the individual level in the West Bandung District was 5.10%, as shown in Table-1.

**Table-1:** Prevalence of brucellosis levels in livestock in West Bandung district.

No	Sub-District	Village	Sample	RBT		CFT	
	
				Positive	%	Positive	%
1	Cisarua	Jambudipa	11	2	18.18	2	18.18
		Pasir Halang	49	3	6.12	3	6.12
2	Lembang	Cibodas	40	0	0.00	0	0.00
		Cibogo	25	2	8.00	2	8.00
		Cikahuripan	45	2	4.44	2	4.44
		Cikidang	15	0	0.00	0	0.00
		Cikole	30	1	3.33	1	3.33
		Jayagiri	25	2	8.00	1	4.00
		Langensari	5	0	0.00	0	0.00
		Lembang	50	4	8.00	3	6.00
		Pagerwangi	30	1	3.33	1	3.33
		Sukajaya	100	4	4.00	4	4.00
		Suntejaya	35	7	20.00	6	17.14
		Wangunsari	25	3	12.00	3	12.00
3	Parongpong	Cihideng	103	3	2.91	2	1.94
Total			588	34	5.78	30	5.10

RBT=Rose Bengal test, CFT=Complement fixation test

**Figure-2:**
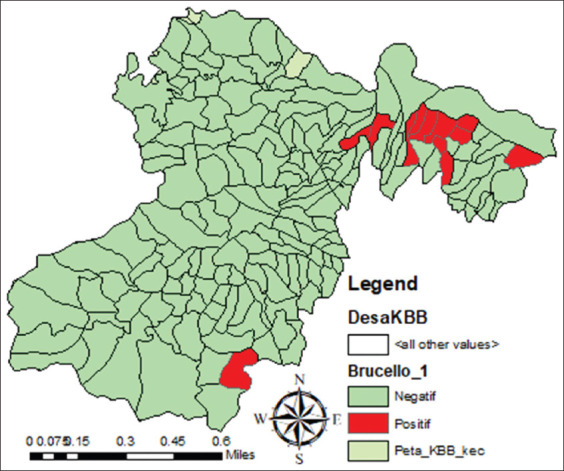
Map of distribution of brucellosis dairy cow in KBB [Source: Map prepared by the corresponding author].

Based on the results, dairy cattle with positive brucellosis were distributed in 12 villages (80%) out of 15 villages (Figure-2).

### Bivariate analysis of risk factors at the individual level in dairy cattle

Risk factors of brucellosis at the individual level of dairy cattle in the West Bandung District are presented in Table-2. A significant association (p<0.05) was shown where the strength of the association (OR) was high for brucellosis in dairy cattle and included the history of abortion with 7-8 months gestation (p=0.000; OR=15.2), the history of abortion (p=0.000; OR=9.9), the history of placental retention (p=0.000; OR=6.6), endometritis (p=0.000; OR=5.5), the history of gestational abortion at the age 4-6 months (p=0.007; OR=3.8), as well as stillbirth (p=0.043; OR=3.0). Furthermore, risk factors in cattle with no association with brucellosis (p>0.05) included the history of abortion factors with 3 months gestation (p=0.363), vaccination (p=0.237), birth cages (p=0.271), calving interval (p=0.771), and the age of >2 years of dairy cattle (p=0.127).

**Table-2:** Univariate analysis and risk factors associated with brucellosis at individual level of dairy cattle in West Bandung district.

No	Variable	Category	CFT		Samples	%	Chi-square	p-value	OR

			Positive	Negative					
1	History of abortion at trimester 3 (7-8 months)								
		1) Yes	16	39	55	9.4	72.1	0.000	15.2
		2) No	14	519	533	90.7			
2	History of abortion								
		1) Yes	19	83	102	17.3	46.6	0.000	9.9
		2) No	11	475	486	82.6			
3	History of placental retention								
		1) Yes	17	92	109	18.5	30.4	0.000	6.6
		2) No	13	466	479	81.4			
4	History of endometritis								
		1) Yes	16	19	35	5.9	24.1	0.000	5.5
		2) No	14	462	476	80.9			
5	History of abortion at trimester 2 (4-6 months)								
		1) Yes	5	28	33	5.6	7.3	0.007	3.8
		2) No	25	530	555	94.3			
6	History of still birth								
		1) Yes	4	27	31	5.2	4.1	0.043	3
		2) No	26	531	557	94.7			
7	History of abortion at trimester I (1-3 months)								
		1) Yes	0	15	15	2.5	0.8	0.363	-
		2) No	30	543	573	97.4			
8	Vaccination								
		1) Yes	28	478	506	86	1.4	0.237	-
		2) No	2	80	82	13.9			
9	Birth Stable								
		1) Yes	11	153	164	27.8	1.2	0.271	-
		2) No	19	405	424	72.1			
10	Calving interval								
		1) ≤12	13	257	270	45.9	0.2	0.771	-
		2) >12	17	301	318	54			
11	Age of Cows (years)								
		1) <2	3	5	8	1.3	2.3	0.127	-
		2) >2	27	553	486	82.6			

CFT=Complement fixation test, OR=Odds ratio

### Brucellosis model in dairy cattle

The result of the multivariate analysis from the logistic regression of brucellosis at the livestock level (Table-3) obtained from the model was CFT cattle=−3,2843+3.4103 the history of abortion in 7-8 months pregnancy +2.5450 abortion in 4-6 months pregnancy +1.8618 cattle with cattle age >2 years −1.0469 the history of calving intervals ≤12 months.

**Table-3:** Logistic regression analysis of brucellosis models at individual level in dairy cattle.

Predictor variables	Coefficient	SE	Coefficient/SE	p-value
Constant	−3.28432	0.83191	−3.95	0.0001
History of abortion at trimesters 3 (7-8 months)	3.41033	0.47463	7.19	0.0000
History of abortion at trimester 2 (4-6 months)	2.54503	0.61417	4.14	0.0000
Age of cow >2 years	1.86185	0.73565	2.53	0.0114
Calving interval ≤12 months	−1.04691	0.59906	−1.75	0.0405
Deviance	176.84			
p-value	1.0000			
Degrees of freedom	583			

SE=Standard error

The results of the logistic regression analysis presented in Tables-3 and 4 showed that the factors responsible for increasing brucellosis in dairy cattle were the history of abortion in 7-8 months pregnancy (β=+3.4103, OR=30.3), abortion in 4-6 months pregnancy (β=+2.5450; OR=12.7), cattle with cattle age >2 years (β=+1.8618; OR=1.2), whereas the factors in association with the reduction of brucellosis in dairy cattle included the history of calving intervals ≤12 months (b=−1.0469; OR=0.34). The model obtained above was relatively accurate as it had Hosmer-Lemeshow goodness of fit test model with a sensitivity value of 66.66% and a specificity value of 83.64%.

**Table-4:** Variable odds ratio values in the brucellosis model at individual level in dairy cattle.

Predictor variables	95% CI		

	Lower limit	Upper limit	Odds ratio
History of abortion at trimester 3 (7-8 months)	11.94	76.75	30.28
History of abortion at trimester 2 (4-6 month)	3.82	42.47	12.74
Age of cow >2 years	0.04	3.66	1.16
Calving interval ≤12 months	0.11	1.14	0.34
Deviance	176.84		
p-value	1.0000		
Degrees of freedom	583		

CI=Confidence interval

## Discussion

### Prevalence of brucellosis in dairy cattle

The prevalence of brucellosis at the dairy cattle level in the West Bandung District was 5.10% (Table-1). With >2% the model was considered high enough for the West Bandung District to be included in the category of heavily-infected areas of brucellosis. This is following the Minister of Agriculture Decree no. 828/KPTS/OT.210/10/1998 article 8 as well as the FAO [26] concerning mildly infected and severely infected areas. A severely infected area is an area with a prevalence of >2%, whereas a mildly infected area is one with a prevalence of <2%. Regions that have a prevalence of >2% in Indonesia, include the Jakarta Province that has a prevalence of 5.9% [27]. Other areas that have a high prevalence of brucellosis are Belu District, and East Nusa Tenggara Province which has a prevalence of 14.5% [28].

### Bivariate analysis of risk factors present at the individual level in dairy cattle

The research results in Table-2 showed that the risk factors in association with the history of abortion at the age of 7-8 months gestation have a strong association (p=0.000) as well as the greatest association strength (OR=15.2) in comparison with other risk factors. These results indicated that the cattle with abortion at pregnancy age of 7-8 months had 15.2 times higher risk of having brucellosis in comparison with other dairy cattle. These observed results were consistent with the risk factor reported in a previous brucellosis study conducted by Ali [29], namely, that history of abortion in the third trimester was statistically significant (p<0.001) with a herd-level brucellosis seropositivity demonstrated by univariate analysis. The main clinical symptom of brucellosis was abortion in the middle and at the end of the pregnancy [9,10,30]. Abortion in cattle usually occurs in the ­third-trimester of the pregnancy due to bacterial growth at the 4^th^ or 5^th^ months of gestation. The maximum production of erythritol in ruminants occurs during pregnancy. Erythritol is a carbon source for Brucella, causing extensive intracellular replication by the Brucella bacteria in the placenta during the late trimester pregnancy [2,31].

Risk factors in association with the history of abortion had a significant association (p=0.000) as well as the strength of the association OR=9.9 showed that the incidence of brucellosis in dairy cattle that had been aborted was 9.9 times higher than that of those which have never had an abortion. This result was consistent with the research of Alhaji *et al*. [20], Tasaime *et al*. [18], and Ndazigaruye *et al*. [21], who reported that the history of abortion was a risk factor for brucellosis. Historical abortion factors were reported to have a significant association with ­brucellosis [21,32,33]. Islam *et al*. [34] stated that history of potential reproductive disorders as risk factors for brucellosis included abortion and placental retention. Moreover, the study of Quin *et al*. [35] and Salmani [36] also proved that abortion is the main symptom of brucellosis.

Risk factors in association with the history of placental retention had a significant connection with brucellosis cases (p=0.000) as well as the strength of the association was OR=6.6. The results of this research indicated the cattle which retained placenta had a 6.6 times higher risk of having an infection of brucellosis compared to that of those cattle which never had placental retention. This perception was consistent with the findings of Deka *et al*. [33] and Gemma *et al*. [37], who reported that a history of placental retention has a significant relationship with the seroprevalence of brucellosis. Moreover, placental retention has a significant relationship with brucellosis and is an important predisposing factor for the development of postpartum uterine infections in dairy cattle [27,38,39]. Placental retention was demonstrated to potentially follow abortion as brucellosis can result in acute or chronic endometritis and terminated permanent sterility in infected cattle [40]. The results of Samaha *et al*. [41] and Tasaime *et al*. [18] showed that a history of placental retention is a factor that increases the incidence of brucellosis in dairy cattle.

The risk factor in association with the history of endometritis had a strong relationship (p=0.000) and the strength of the association was OR=5.5, meaning that the cases of brucellosis in dairy cattle having endometritis were 5.5 times higher compared to those in case of cattle without endometritis. This finding was consistent with the observation of Patel *et al*. [32] who stated that the risk factors, including the history of metritis/endometritis had statistically significant effects on the prevalence of brucellosis. Infectious reproductive diseases were previously reported to cause endometritis, embryo death, infertility, retained placenta, central nerve damage from the fetus, and sterility in bulls [42]. High rates of abortion and reproductive disorders, including metritis, were also reported in association with seropositive brucellosis [43-45].

Risk factors in relation with the abortion history of the gestational age of 4-6 months also had a strong association with cattle brucellosis (p=0.007) with a strength of association OR=3.8 meaning that the cattle which had an abortion in 4-6 months of pregnancy have a 3.8 times higher risk of having Brucellosis than those dairy cattle which have never experienced pregnancy abortion at the age of 4-6 months. This is in line with OIE [4] which states that brucellosis infection in pregnant cattle will cause placentitis; therefore, abortion occurs at the gestational age between 5 and 9 months.

The research results showed that the risk factor in association with the history of stillbirth has a strong association (p=0.043) and the strength of the association was OR=3.0. These results indicated that those dairy cattle which had a history of stillbirth have a 3.0 times higher risk of having brucellosis than those cattle that have never experienced stillbirth. Brucellosis in cattle is a chronic infectious disease characterized by the birth of a weak or dead calf [46,47], while this reproductive disorder could be a consequence caused by placenta retention and endometritis [48]. Risk factors, such as stillbirth, have a statistically significant effect on the prevalence of brucellosis [32,33].

Risk factors for the history of abortion at the age of 1-3 months gestation had no association (p=0.363) with the brucellosis of dairy cattle. This was due to that erythritol as a growth agent is needed for *B. abortus* to begin to be produced at the middle age of (4-5 months) pregnancy. Erythritol production was reported to be the highest during mid-pregnancy [49]. Erythritol production increases dynamically following the increased vulnerability to *Brucella* colonization that occurs during the second half of pregnancy [50]. Erythritol production in the middle of the pregnancy causes abortion in the latter stage of pregnancy; therefore, it is characterized as the main symptom of abortion in the final pregnancy stage. One of the main symptoms of brucellosis in farm animals is abortion in the advanced stages of the third trimester of pregnancy. Flocks that included animals with a history of abortion especially in the third trimester were more likely to be seropositive. Similar results were also found in Uganda and Kenya [51].

Other risk factors, including vaccination, showed no relationship (p=0.127) with brucellosis in the West Bandung District. Vaccination is one of the policy programs implemented by the government of the West Bandung District as an effort to prevent brucellosis due to its prevalence of >2%. According to the OIE [4]), an area with a high prevalence of brucellosis (>2%) is categorized as endemic and vaccination is suggested for its control. A vaccination program was applied to dairy cattle in the West Java District; however, brucellosis cases have remained high as the effectiveness of vaccination is still low due to its unknown level of protection. The coverage of dairy cattle vaccination was 86.05% that was considered high; however, the level of protection was unknown. Vaccination should ideally protect vulnerable populations that are at high risk of infection [52] with an aim to reduce vulnerable individuals in the population. The success of each vaccination program depends primarily on the effectiveness of the vaccine used and its coverage in the target population [2,53,54]. Even though the S19 vaccine has a better efficiency for protection, in the West Bandung District the RB-51 vaccine type is used. The RB-51 strain has a very good record of stability in comparison with that of the S19, and it can differentiate among infected and vaccinated animals (property) when used in a cattle population. The RB-51 strain was resistant to rifampicin, an important antibiotic used in the treatment of brucellosis. But recently, it has been reported that cattle vaccinated with RB-51 in the Greater Yellowstone Area in the USA were still vulnerable to brucellosis [55,56]. The RB-51 vaccine was reported to show low protective effectiveness [57].

### Multivariate analysis of model brucellosis in dairy cattle

The history of abortion for cattle with 7-8 months of gestational age showed to increase brucellosis (β=+3.4103) in dairy cattle. One of the main symptoms of brucellosis in farm animal herds was the occurrence of abortion in the latter stage of pregnancy (third trimester). Abortion in cattle brucellosis generally occurs from the gestational age of 6-9 months [4,57]. Flocks that had animals with a history of third-trimester abortion were found to be higher in seropositive brucellosis [32,33,58]. The third-trimester abortion history [59] as well as the abortion history [60] was found to be significantly associated with bovine brucellosis. Abortion in cattle caused by *Brucella* would usually occur at the gestational age between 5 and 8 months [11]. The host mechanism responsible for increased susceptibility to brucellosis infection in advanced pregnancy is related to the differential susceptibility of the placental trophoblast during the middle and late stages of pregnancy. High concentrations of erythritol in the uterine tissue as well as the ability of *B. abortus* to use this rare sugar are pathogen determinants in cattle [2,61].

Cattle that have a history of abortion at the gestational age of 4-6 months were also found to increase brucellosis (β=+2.5450) in dairy cattle. Abortion is the main symptom in association with the incidence of bovine brucellosis as the host that is prone to brucellosis is in pregnancy [62]. Bovine brucellosis is a chronic infectious disease characterized by 5-7 months of long-term abortion [2,4,63], the birth of weak or dead calves from pregnant dairy cattle [7,48,62,64], and fertility disorders caused to maintain the placenta and the endometritis [48,63]. Reproductive disorders, especially repeated breeding and previous abortion history, were found to be significantly associated with bovine brucellosis [65].

Age factors >2 years were found to be associated (p=0.0114) with an increased number of cases of brucellosis (β=+1.8618) in dairy cows. Adult cows are more ready to get pregnant as their reproductive organs are mature. During pregnancy, erythritol will be produced as a growth agent needed by *B. abortus*. Besides, several studies mentioned that age is a risk factor for brucellosis and the prevalence of the disease is directly proportional to the older age [58,66]. Erythritol is a preferential carbon source for most *Brucella*, a group of facultative intracellular bacteria that cause zoonosis around the world. It is abundant in ruminant and pig genital organs and plays a role in some of the characteristics of *Brucella* genitalism [67]. *Brucella* spp. presented tropism to the reproductive tract due to the production of erythritol, a 4-carbon sugar produced in ruminant fetal tissue that stimulates *Brucella* growth. According to Coelho *et al*. [2], the prevalence of brucellosis is higher in adult animals than in young animals.

The calving interval factor of ≤12 months was demonstrated to have the effect of decreasing brucellosis (β=+1.0469) in dairy cattle. The calving interval is a combination of the pregnancy time and free time. A good calving interval is ±365 days [68]. Moreover, the ideal distance between births is 12 months [58]. The results of a previous study conducted by Ndazigaruye *et al*. [21] stated that the calving interval of animals with positive brucellosis for 65.8% of respondents was longer than 1 year. Long calving intervals for a cow may be due to a disruption in its reproductive status and could be an indirect effect of abortion. Abortion can be caused by various traumatic factors, the lack of nutrition, as well as infections that disrupt the reproductive function [30].

## Conclusion

The prevalence of brucellosis at the individual level of dairy cattle in the West Bandung District was 5.10%. Risk factors of brucellosis in dairy cattle based on bivariate and multivariate analysis that had a strong association were the history of abortion at the age of 7-8 months of gestation, the history of endometritis, the history of placental retention, the history of abortion at the age of 4-6 months of pregnancy, and the history of stillbirth. Risk factors that increase the cases of brucellosis based on multivariate analysis are the abortion history of pregnancy at the age of 7-8 months, the abortion history of pregnancy at the age of 4-6 months, and the age of >2 years, while the calving interval history factor of ≤12 months was found to have a negative effect and the potential to reduce the case of brucellosis in dairy cows.

## Authors’ Contributions

BS, TAK, AP and SS participated equally in the study plan and design. YY performed in the study design, fieldwork, collected the samples, and prepared the manuscript as a part of his research. BS and TAK carried out proofreading and made critical comments on this manuscript. All authors read and approved the final manuscript.
